# A cosmetic treatment based on the secretion of *Cryptomphalus aspersa* 40% improves the clinical results after the use of nonablative fractional laser in skin aging

**DOI:** 10.1111/jocd.13052

**Published:** 2019-06-21

**Authors:** M. Teresa Truchuelo, Maria Vitale

**Affiliations:** ^1^ Vithas Nuestra Señora de América Hospital Madrid Spain; ^2^ Medical Department of Cantabria Labs Madrid Spain

**Keywords:** aging, cosmetic, laser, laser recovery, rejuvenation, treatment

## Abstract

**Introduction:**

The main purpose of this study was to evaluate whether the application of a cosmetic treatment based on the secretion of *Cryptomphalus aspersa* (SCA) enhances the clinical results, tolerance, and skin regeneration after nonablative laser treatment in patients with moderate photoaging.

**Methods:**

Randomized, double‐blind, split‐face trial in 20 patients with moderate aging. Two sessions with fractional nonablative laser were performed, and the cosmetic treatments (SCA 40% on one hemiface and vehicle on the other) were applied immediately after laser session and daily during the study (28 days). Tewameter, Cutometer, Visioscan, VisioFace, photography, dermoscopy, and clinical evaluation were assessed. Side effects were also evaluated.

**Results:**

A significant decrease in the density of microcolumns (25%, 71%, 32%, and 61% less density, respectively, at T3 *P* = 0.008, T7 *P* = 0.002, T22 *P* < 0.001, and T24 *P* < 0.001) was observed on the side treated with SCA compared to the vehicle‐treated side. Cutaneous elasticity, area of wrinkles, and hydration on the SCA‐treated side also showed a significant improvement compared to the vehicle‐treated side. Both the researcher and patients observed a significant improvement on the side treated with SCA compared to the vehicle‐treated side. Significantly fewer side effects (erythema, burning, and dryness) were also detected.

**Conclusion:**

A cosmetic product with SCA 40% applied immediately after laser and for a period thereafter enhances and accelerates repair of damage produced by the laser and significantly reduces related adverse effects. In addition, SCA treatment could improve clinical results. In conclusion, we suggest that SCA enhances the effectiveness of laser in the treatment of cutaneous aging.

## 1 BACKGROUND

Skin aging is a physiological process determined by endogenous mechanisms and external environmental aggression.^1^ Exposure to the sun, pollution, and tobacco triggers molecular processes that damage the skin structure, leading to an aged skin appearance. Other, less well‐studied factors (temperature, lack of sleep, nutrition, and stress) also promote cutaneous aging.^2^ The subsequent activation of nuclear factor‐κB (NF‐κB) and factor activating protein‐1 (AP‐1) through different mechanisms induces the expression of matrix metalloproteinases, accelerating matrix degradation.^3^, ^4^, ^5^, ^6^

Skin aging treatments include different approaches. Among them, nonablative fractional laser technology consists in applying the laser energy that penetrates the skin forming small microcolumns (MTZs: microscopic thermal zones) leaving part of the skin intact where heat accumulates and biological effects occur: increased vascularity, greater supply of nutrients, and stimulation of the synthesis of collagen. The undamaged skin acts as a reservoir of that heat and contributes to create new skin improving the aesthetic appearance of the skin.^7^, ^8^, ^9^ Dermal activation induces new collagen with consequent clinical improvement.^10^, ^11^ With the use of nonablative fractional laser, the recovery time is faster and side effects are fewer when compared to ablative fractional laser.^8^ In addition, nonablative fractional lasers are successful in increasing the penetration of cosmetics and drugs applied topically after the laser session (laser assisted drug delivery).^8^, ^12^, ^13^, ^14^, ^15^, ^16^

In the present study, we applied a cosmetic product containing SCA 40% immediately after laser treatment. SCA 40% is a cosmetic ingredient obtained from gastropods of the family *Cryptomphalus aspersa*. The analytical profile of the secretion indicates a richness of proteins of low molecular weight, similar to the fibroblast growth factor; antioxidant enzymes; and glycosaminoglycans.^17^, ^18^ Through numerous studies, SCA demonstrated its ability to induce cutaneous regeneration and antioxidant efficacy through superoxide dismutase and glutathione transferase activity.^19^, ^20^ Several in vitro and clinical studies have shown that SCA stimulates the proliferation and migration of fibroblasts and keratinocytes accelerating the process of wound healing.^21^, ^22^, ^23^, ^24^

## 2 AIMS

The main objective of this study was to assess the efficacy of the cosmetic product under study (SCA 40%) in inducing faster skin recovery after performing a nonablative fractional laser treatment in patients with moderate photoaging. As secondary objectives, we studied the synergistic effectiveness in improving the laser procedure outcome and the subjective evaluation of photoaging improvement perceived by patients and the investigator.

## 3 PATIENTS/METHODS

This was a prospective, randomized, double‐blind, split‐face study where SCA 40% was applied to one side of the face *versus* vehicle on the other in 20 patients. The active treatment (SCA) or the vehicle was randomly assessed to be applied in the right hemiface (RH) or the left hemiface (LH), the patient acting as his/her own control. Patients with moderate facial skin aging were recruited (minimum assessment of 3 on the scale of Rao‐Goldman), and featured the following inclusion criteria:

- Aged 45‐65 years.
- Absence of other skin treatments during the last 3 months
- Absence of desire for pregnancy during the study period and use of contraceptive methods
- No concomitant diseases
- Avoidance of other topical or concomitant systemic products
- Desire to treat their skin aging signs with laser
- Understanding and signature of informed consent
- Absence of allergy to the components of the study product
- No use of regular cosmetics products for one week before beginning the study.

The study followed the tenants of the Declaration of Helsinki.

### 3.1 Treatment regimen

The active formula included SCA 40%. Treatments were performed with nonablative fractional laser, and two laser sessions were performed, the first at T0 and the second at T21. A 1540‐nm Erb‐glass laser from the ICON platform of Palomar Technologies (Cynosure, Madrid, Spain) was used at 50 mj of energy and pulse duration of 15 ms. Three passes were made on the patient's face in each of the sessions. The product was applied immediately after the laser treatment session and during the subsequent 28‐day study period. The product was applied every 12 hours for the first 7 days after performing the laser session and then every 24 hours until the next laser session. After the second session, the product was applied again every 12 hours during the remaining 7 days of the study. The patients also applied topical photoprotection (Heliocare 360 Mineral Tolerance Fluid) in the morning from T8 to T21. Eight visits were performed: T0 (first laser session), T1d (24h), T3d (72h), T7d, T21d (second laser session), T22d (24h), T24d (72h), and T28d.

### 3.2 Clinical assessment

At all visits, clinical assessment, photography, and transepidermal water loss (TEWL) as hydration measurement were carried out. The assessment of skin elasticity and firmness (Cutometer) and the quantitative assessment of wrinkles and skin texture were performed at T0 and T28.

#### 3.2.1 Instrumental evaluation

*Micro‐dermatoscopic photography*: dermatoscopic evaluation of the density of the microcolumns (0‐3): 0: zero density; 1: light density; 2: moderate density; and 3: intense density. Camera Medicam® 1000, from FotoFinder Systems, Inc (7100, Columbia, Maryland, USA).
*Tewameter® TM300 (Courage & Khazaka electronic GmbH, Germany)*: quantitative evaluation of the transepidermal water loss through the stratum corneum, as a measure of skin hydration and integrity of the skin barrier.
*Cutometer® Dual MPA 580 (Courage & Khazaka electronic GmbH, Germany)*: instrumental quantitative evaluation of the mechanical and viscoelastic parameters of skin firmness (R0) and elasticity (R2).
*Visioscan® VC 98 USB (Courage & Khazaka electronic GmbH, Germany)*: determination of effectiveness against wrinkles through topographic analysis of epidermis at T0 and T28.
*VisioFace 1000® (Courage & Khazaka electronic GmbH, Germany)*: determination by macrophotography of the effectiveness against wrinkles at T0 and T28. The image analysis included the value in pixels (length and area of the wrinkle).

#### 3.2.2 Subjective evaluations

*Severity of adverse effects* after laser in RH and LH: erythema (0‐3), edema (0‐3), burning (0‐3), tightness (0‐3), and others. 0: null; 1: mild; 2: moderate; and 3: intense.
*Severity of photoaging perceived by the researcher* in RH and LH according to the validated Rao‐Goldman 5‐point scale (RGWS): (1) without wrinkles, (2) visible but fine wrinkles, (3) moderately deep wrinkles, (4) deep wrinkles with defined edges, and (5) very deep wrinkles with defined grooves. For its evaluation, macrophotography was carried out.
*Improvement perceived by the researcher*: IGA (−2 to 3) in RH and LH: 0: none; 1: minimal improvement; 2: moderate improvement; 3: intense improvement; −1: minimum worsening; and −2: great worsening. It was evaluated at 3, 21, and 28 days after laser treatment.
*Improvement perceived by patients*: PGA (−2 to 3) in RH and LH: 0: none; 1: minimal improvement; 2: moderate improvement; 3: intense improvement; −1: minimum worsening; and −2: great worsening. It was evaluated at 3, 21, and 28 days after laser treatment.

### 3.3 Statistical study

For the efficacy variables of ordinal nature, a nonparametric Wilcoxon test was performed. For the quantitative variables, mixed linear models (MLM) and generalized estimation equations (GEE) were used. As descriptors, the most usual values of centralization, dispersion, and position were used in all cases: mean, standard deviation, and median. A difference between the hemifaces was considered statistically significant at *P* ≤ 0.05. R software was used to perform the statistical calculations.

## 4 RESULTS

We included 20 subject volunteers, and there were not dropouts or exclusions.

**Figure 1:**
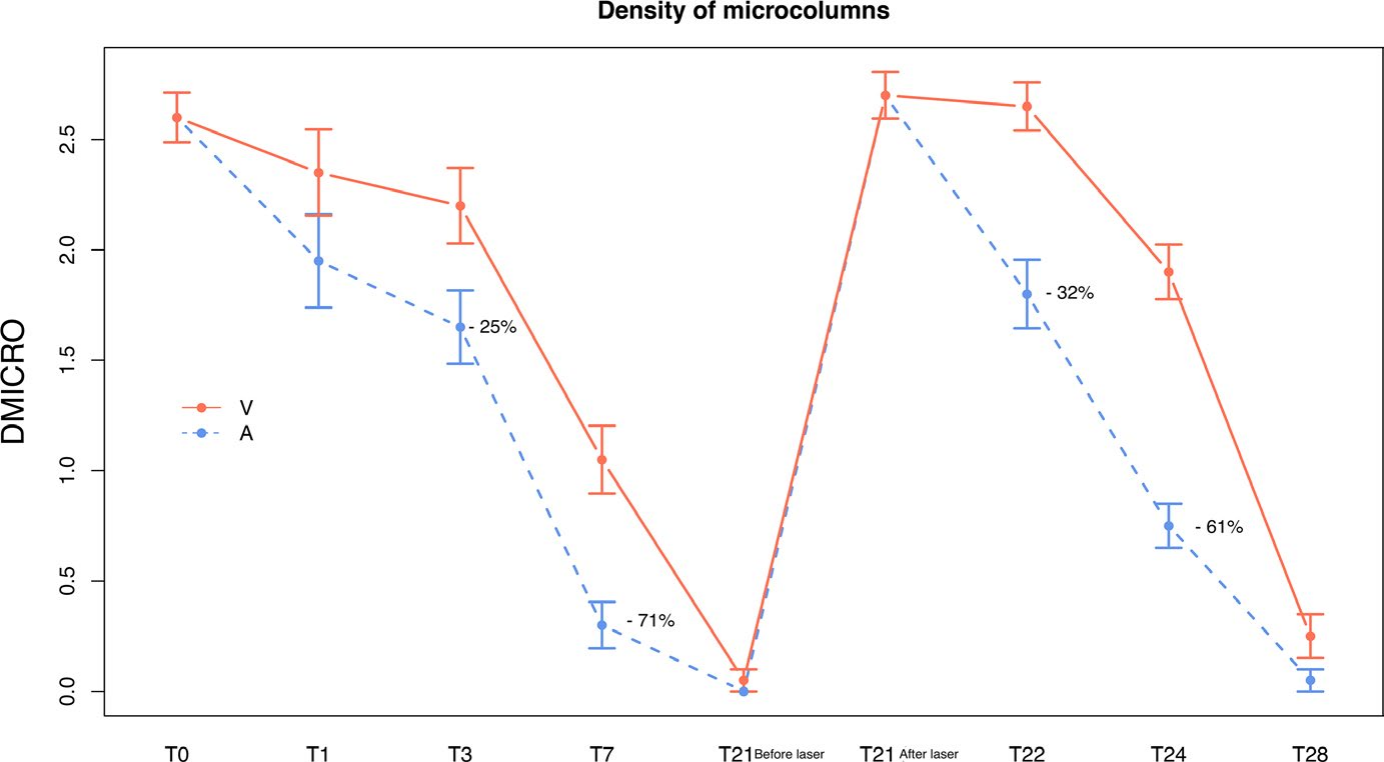
Recovery of density of microcolumns after laser in both hemifaces, faster with SCA. Significant percentages of decrease with SCA treatment are shown. A, active; V, vehicle; T, time

**Figure 2:**
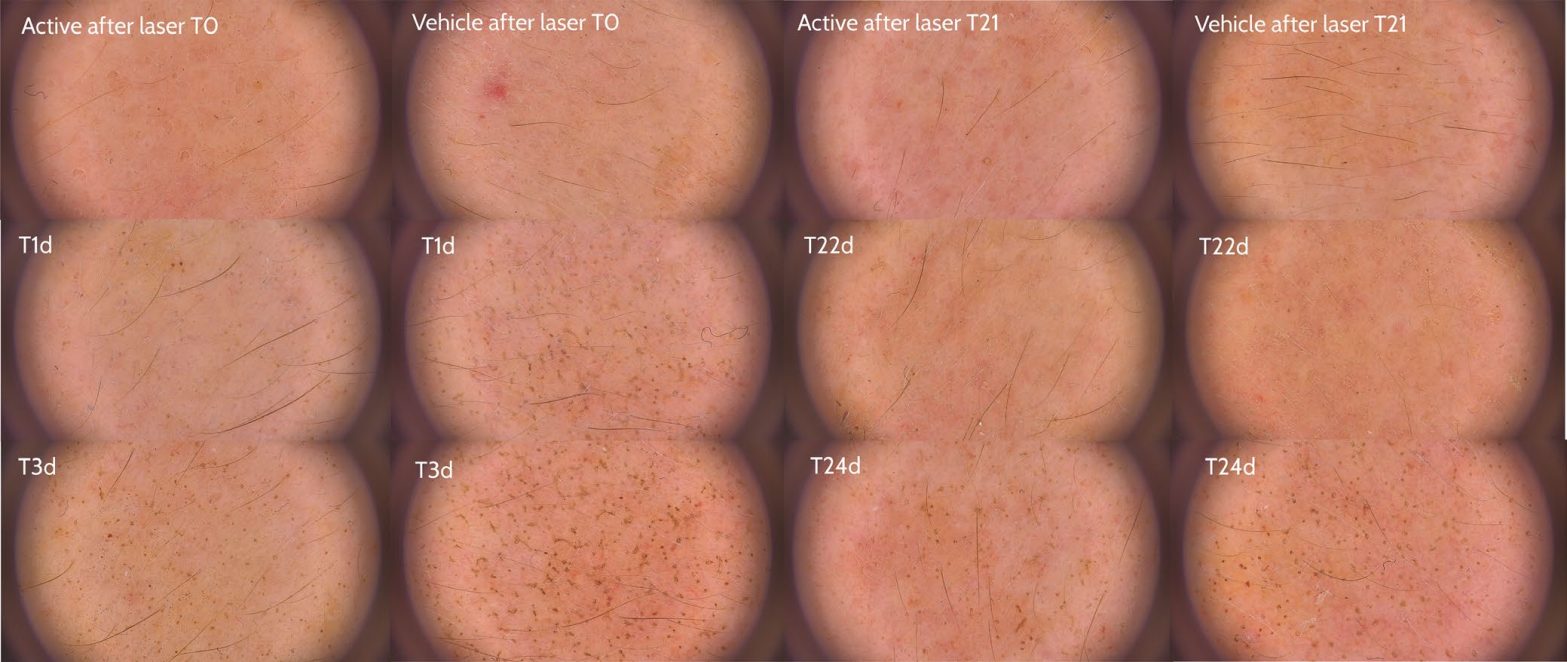
Evolution of microcolumn density with the active (left) and vehicle (right) treatments in Patient 15

The microcolumn density induced by the laser according to dermoscopic evaluation decreased significantly faster on the side treated with the active ingredient, compared with the vehicle‐treated side (T3 *P* = 0.008; T7 *P* = 0.002; T22 and T24 *P* < 0.001) (Figure 1). The difference was even more marked at 72 hours after the second laser treatment (T24). This finding suggests that continuous treatment with SCA 40% between laser sessions increases skin regeneration even more after the second laser treatment (Table 1, Figure 2).

**Table 1:** Moderate or intense density of microcolumns at dermoscopic evaluation (FotoFinder Systems) at T3, T22, and T24

Time	Active (%)	Vehicle (%)	*P* value
T3	45	85	0.0203
T22	65	100	0.0125
T24	10	70	0.0004

**Figure 3:**
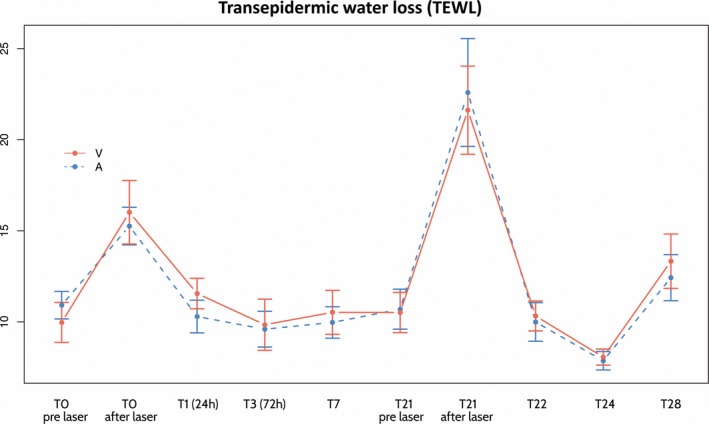
Graph with the summary of evolution of transepidermal water loss (TEWL) on the side treated with the active (A) and vehicle (V). A peak of increase was observed at T0 and also at T22 immediately after the second laser session. T, time

Regarding TEWL, both hemifaces showed a significant increase (*P* < 0.001) in the measurements performed immediately after both laser sessions (T0A), showing the epidermal alteration induced by laser. However, 24 hours later (T1) a significantly less TEWL was detected on the active‐treated side versus the vehicle‐treated side, with a difference between treatments of 11% (*P* = 0.020). These differences between the sides equalized at T3 (Figure 3).

**Figure 4:**
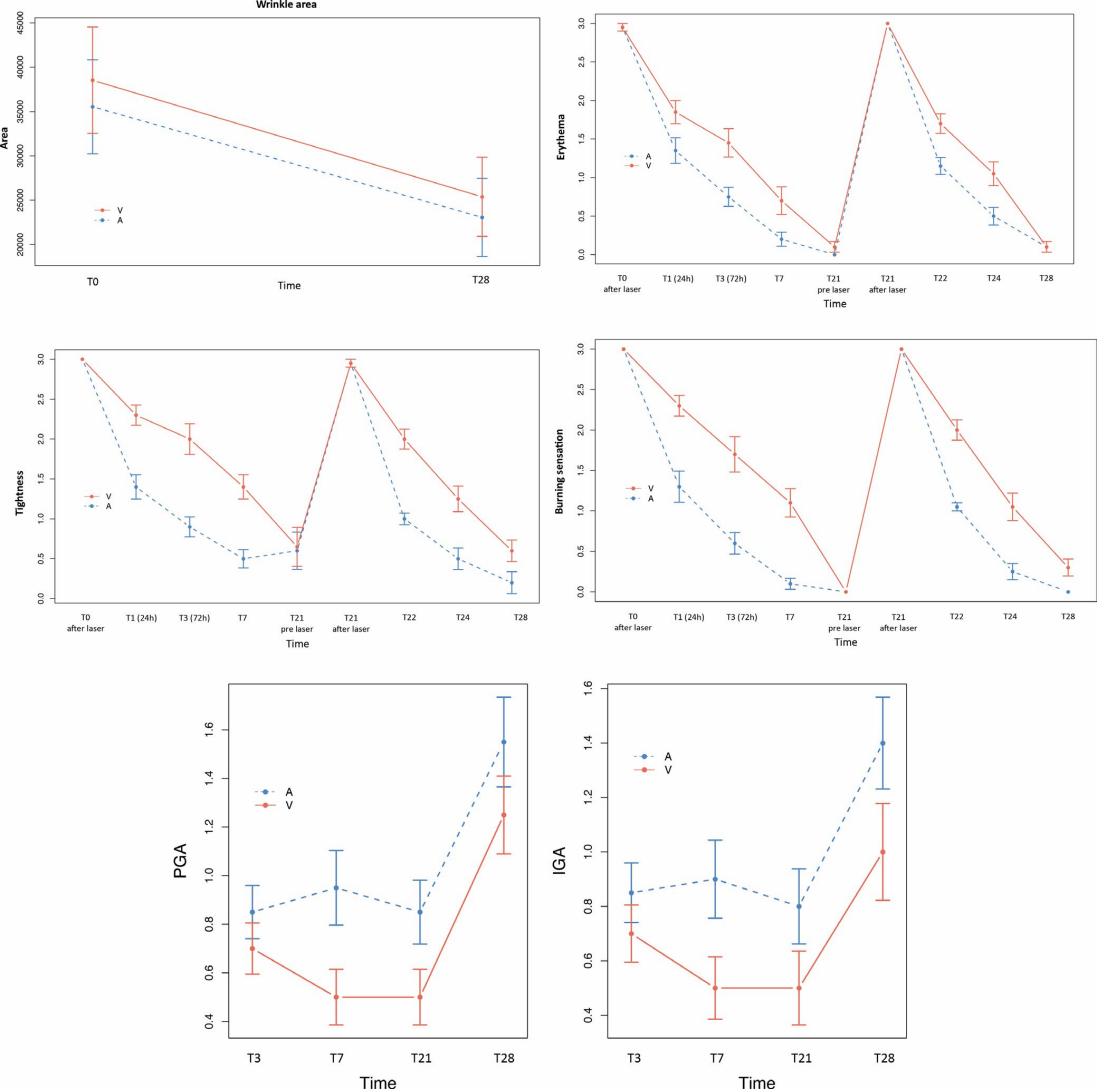
A significantly better improvement in wrinkle area was detected on the active‐treated side (A) compared to the vehicle‐treated side (V). On the right side and below the previous image, we show the significantly greater improvement in erythema, tightness, and burning sensation in the SCA‐treated hemiface (A) compared with the vehicle‐treated hemiface (V). The lower graphs show the improvement perceived by the patients (PGA) and the investigator (IGA). At all time points, the improvement was greater on the active‐treated side (A) compared to the vehicle‐treated side (V), reaching significant differences for PGA at T7 and for IGA at T7 and T21. T, time

Regarding the side effects, the following were observed. There was a significantly greater decrease in erythema on the active‐treated side vs. the vehicle‐treated side at T3, T7, T22, and T24 (*P* = 0.036, *P* = 0.036, *P* = 0.009, and *P* = 0.009, respectively). Burning sensation increased immediately after the laser treatment. With respect to the vehicle, the reduction in burning sensation with SCA was significant and 43% greater at all visits except T22. The decrease in the tightness sensation was significantly greater in the side treated with the active compared to the vehicle‐treated side at T3, T7, T22, and T24 (*P* = 0.001, *P* = 0.001, *P* < 0.001, and *P* = 0.004) (Figure 4). Nonsignificant differences were detected in edema between hemifaces.

Other objective variables studied showed a significant improvement in elasticity in the SCA‐treated hemiface (reaching an increase of 11%) at the end of the study (*P* = 0.037), whereas no significant changes were detected in the vehicle‐treated hemiface. No significant differences were detected in firmness either between treatments or with respect to T0. There was a significantly greater mean decrease in the area of wrinkles on the side treated with SCA (*P* = 0.04), with a 38% reduction in 90% of patients on the SCA‐treated side vs only a 20% reduction in 65% of patients on the vehicle‐treated side (Figure 4).

The subjective evaluation using the Rao‐Goldman scale did not show significant difference between hemifaces. On the other hand, both researcher and patient global assessments indicated a significantly better improvement on the side treated with SCA: IGA at T7 showed an 80% greater improvement on the active‐treated side vs the vehicle‐treated side (*P* = 0.032). PGA at T7 and T21 showed a 90% and 70% difference, respectively, in the improvement assessment in favor of the active (*P* = 0.032 and *P* = 0.042) (Figure 4).

## 5 DISCUSSION

Fractional laser induces a controlled damage consisting of microcolumn generation in the skin. A significantly lower microcolumn density was quickly detected at the SCA‐treated side, improving recovery time when compared to vehicle. In addition, we suggest that cutaneous regeneration could be accelerated with the use of SCA, as we observed significantly lower microcolumn density just after 24 hours (T22) of the second procedure, faster than with the first laser when significant improvement was after 72 hours (T3).

The TEWL levels reflect the state of the barrier function. Although nonablative laser did not produce direct damage to the stratum corneum, significant increases in TEWL were detected in both hemifaces after laser treatment. Over time, the barrier function progressively recovered in both hemifaces, and with it, TEWL progressively decreased. Twenty‐four hours after laser treatment, a significantly greater reduction in TEWL was achieved on the SCA‐treated side. Moreover, at T1 (24 hours postlaser) the side treated with vehicle continued worsening (TEWL increased by 16%), while the side treated with the active had already recovered and even improved when compared to prelaser levels (TEWL −6%, not significant). These findings support the speed of action in terms of skin barrier regeneration on the SCA‐treated side. The reduction in microcolumn density (significant from T3) correlates directly with the recovery of the barrier function. These data agree with the in vitro and clinical studies that showed the ability of the growth factors present in SCA to stimulate both the proliferation and migration of fibroblasts and keratinocytes (even in cells in the senescence pathway), as well as the ability to regenerate wounds.^17^, ^18^, ^19^

A formulation that reduces adverse effects induced by laser (edema, erythema, burning, and tightness) implies greater treatment tolerability and faster healing. In the present study, erythema, burning, and dryness disappeared significantly faster on the SCA‐treated side. This would be the result of the regenerative and antioxidant activity already observed with SCA in both in vitro and clinical studies.^19^, ^20^

Significant differences in elasticity were detected with SCA treatment when compared to baseline and the vehicle treatment; however, no changes in firmness were detected. We hypothesize that a firmness effect might be detected more in the long term (from the 2nd to 3rd month of follow‐up) when the result of fibroblast stimulation and skin remodeling may be more noticeable. The improvement obtained on the side treated with vehicle is the result of the stimulus induced by the nonablative fractionated laser. In conclusion, the significantly greater improvement in elasticity in the SCA‐treated hemiface indicates that SCA has a synergistic effect and enhances the elasticity that lasers generate. This observation is supported by previously published studies where SCA demonstrated the ability to induce improvement in the dermal matrix.^20^, ^22^ The significant improvement in wrinkles in both hemifaces compared to baseline and the significantly greater improvement with SCA vs vehicle show that wrinkle reduction is due mainly to the effect of the fractionated laser and secondarily to the synergistic effect of the use of SCA during the entire treatment period. The growth factors and glycosaminoglycans present in the active ingredient SCA have shown in previously published studies this dermal stimulus, which induces improvement in the area affected by wrinkles.^24^

Regarding PGA and IGA, a week after the laser treatment, both the researcher and patients found a significantly greater improvement on the side treated with the active, suggesting that the application of SCA improves global perception of improvement. All results obtained are coherent, with the correlation between improvement in the recovery of microcolumns, reduction in TEWL, and reduction in adverse effects such as erythema, burning, and tightness.

In conclusion, the application of a cosmetic product formulated with SCA 40% immediately after laser treatment and during the following days accelerates recovery from the damage produced by the laser and significantly reduces associated adverse effects. In addition, it is suggested that SCA enhances the effectiveness of the laser in the treatment of aging in terms of elasticity and reduction in wrinkle area.

## CONFLICT OF INTEREST

Dra Truchuelo is scientific adviser to Cantabria Labs.
